# Unraveling the Influences of Sodium, Potassium, Magnesium, and Calcium on the Crystallization Behavior of Lactose

**DOI:** 10.3390/foods12244397

**Published:** 2023-12-07

**Authors:** Rangani Wijayasinghe, Todor Vasiljevic, Jayani Chandrapala

**Affiliations:** 1 Advanced Food Systems Research Unit, Institute of Sustainable Industries & Liveable Cities, College of Health and Biomedicine, Victoria University, Melbourne, VIC 8001, Australia; rangani.wijayasinghe@live.vu.edu.au (R.W.); todor.vasiljevic@vu.edu.au (T.V.); 2 Biosciences and Food Technology, School of Science, RMIT University, Melbourne, VIC 3083, Australia

**Keywords:** lactose, crystallization, acid whey, salts

## Abstract

The inability of lactose to properly crystallize due to the presence of high amounts of salts poses significant hurdles for its downstream processing with some dairy waste streams such as acid whey. This study aimed to investigate the physicochemical and thermal behaviors of lactose in the presence of cations commonly present in acid whey. A model-based study was conducted, utilizing various cations (Mg, Ca, K, and Na) at concentrations (8, 30, 38, and 22 mM, respectively) that are typically found in acid whey. The research experiments were conducted using a factorial design. The thermal analysis of concentrated solutions revealed augmentation in the enthalpy of water evaporation in the presence of individual cations and their combinations in comparison with pure lactose (698.4 J/g). The degree of enthalpy increased following the order of Na^+^ (918.6 J/g), K^+^ (936.6 J/g), Mg^2+^ (987.0 J/g), Ca^2+^ (993.2 J/g), and their mixture (1005.4 J/g). This resulted in a substantial crystal yield decline in the exactly reversed order to that of the enthalpy. The greatest decline was observed in the presence of the salt mixture (63%) followed by Ca (67%) compared with pure lactose (79%). The yield reduction was also inversely related to the solubility of lactose. The presence of divalent cations appeared to play a role in the isomerization of lactose molecules observed using DSC and XRD diffractograms according to the disappearance of peaks related to β lactose. The effect of salts on the crystallization of lactose was a combination of cation–lactose interactions, changes in the solubility of lactose, ion–dipole interactions between water and cations, and changes in the structure of water molecules. By deviating the composition of acid whey, the crystallization of lactose can be enhanced, leading to the improved downstream processing of acid whey.

## 1. Introduction

The process of lactose crystallization in foods is highly complex and involves a multitude of factors that impact the ultimate product quality and processability. Proper control over the crystallization process is essential for preserving the quality, extending the shelf life of specific food products, and increasing downstream processability during the processing of different food streams or even manufacturing of certain food products. For example, lactose crystallization is the first processing step in the separation of lactose from whey solutions originating from cheese and yogurt production. These by-products are commonly referred to as serum that remains subsequent to the coagulation and filtration of milk [1].

Industrially, any whey is concentrated before lactose crystallization to achieve 60–65% of total solids, out of which 50–55% is composed of lactose. This concentrated whey is subjected to spray drying, resulting in the production of powders that find extensive usage in diverse food applications [2]. However, the processing of acid whey, which originates as a by-product of acid-coagulated products such as Greek yogurts or soft cheeses, is widely regarded as a challenging task, primarily due to the inability of lactose to undergo crystallization. This results in lactose persisting in its amorphous state during the concentration process, thereby impeding subsequent processing steps. Due to hindered lactose crystallization in acid whey, the yielded spray-dried powders are hygroscopic with a high propensity to sticking and caking [3]. More recently, it was shown that the presence of lactic acid induced the formation of a strong hydration layer surrounding lactose molecules, thereby hindering water removal and inhibiting lactose crystallization [4] A considerable amount of literature has been published on lactose behavior in the presence of different additives such as lactic acid [5], proteins [6], and calcium [7,8,9]. Previously, it was established that the salts present in a lactose solution can decrease or increase the growth rate of lactose crystals [10], which was also clear from a previous report [11] showing the rate of lactose crystal growth after the addition of potassium (K) as KCl. Salt-induced changes in lactose crystallization can also partially be attributed to changes in pH, as this affects the mutarotation of lactose, and a reduction in pH reduces the crystal growth [12].

Cationic impurities in lactose solutions also govern lactose crystallization in a direction that is dependent on the concentration and the type of the cation present [8]. It was hypothesized that alterations in the solubility of lactose are responsible for the varying crystallization patterns observed in the presence of mineral impurities [13]. For instance, ionic Ca^2+^ ion has been found to play a significant role in the organization of water structure. Ca^2+^ is a divalent ion that possesses a robust electric field, which acts as a promoter of water structuring. The resulting structural changes in water molecules can have a direct impact on the solubility of lactose, which is a crucial factor governing lactose crystallization [14]. Similarly, the inclusion of sodium phosphate has been observed to decrease lactose solubility [11] due to the high charge density of water-structure-making ions, leading to the reorientation of water molecules, which, in turn, reduces the solubility of lactose. On the other hand, the addition of potassium phosphate has been found to increase lactose solubility [15], which is attributed to the water-structure-breaking capability of potassium. However, lactose’s behavior in the presence of different impurities differs according to various phases of the crystallization process, including concentrated solution [4], spray-dried powders [5,16], and crystals [9], due to the different interactions taking place within the system.

Despite the existence of a significant body of literature on this topic, the behavior of lactose in the presence of cations remains a complex and poorly understood phenomenon. As such, more systematic studies on the impact of cations present in acid whey on the crystallization behavior of lactose are needed. Thus, the present study evaluated the physicochemical and thermal behaviors of lactose affected by different cations (Mg^2+^, Ca^2+^, K^+^, and Na^+^) at concentrations frequently encountered in acid whey waste streams, either individually or in combination. In the context of utilizing salts as additions of various cations, a consistent anion in the form of chloride (Cl^−^) was upheld to achieve homogeneity in the impact of the anion in this study.

## 2. Materials and Methods

### 2.1. Materials

Lactose powder and analytical-grade salts (KCl, NaCl, MgCl_2_, and CaCl_2_) were obtained from Murray Goulburn Co-operative Co., Ltd. (Brunswick, Melbourne, Australia) and Sigma–Aldrich Pvt. Ltd. (Macquarie Park, Sydney, Australia), respectively. All solutions were prepared fresh using Milli-Q water at all times.

### 2.2. Preparation of Lactose Solutions

Lactose solutions were prepared to mirror the concentration of salts and lactose present in acid whey (Table 1). Initially, lactose powder was dissolved in Milli-Q water at 70 °C to achieve a concentration of 40 g of lactose per 100 g of solution. The solution was gently agitated with a magnetic stirrer to accelerate the dissolving process. Any undissolved lactose particles were removed by filtering the solution through 0.45 μm Whatman filter paper. Stock solutions of 5 M composed of calcium chloride (CaCl_2_), magnesium chloride (MgCl_2_), potassium chloride (KCl), and sodium chloride (NaCl) were prepared individually. The filtered lactose solution was divided into five parts, and calculated amounts of salts were added to mimic the concentrations of salts present in acid whey to obtain lactose solutions containing the following levels of salts: 38 mM K, 30 mM Ca, 22 mM Na, 8 mM Mg, or a combination of the aforementioned salts at their respective concentrations, as shown in Table 1. A lactose solution containing no added salts was used as the control sample (PL). All the solutions were stirred using a magnetic stirrer to obtain homogenous samples.

Lactose solutions with and without added salts were subsequently concentrated using a rotary evaporator at 55–60 °C to achieve ~50% (*w*/*w*) total solids, based on refractometry measurements (Atago Abbe, Tokyo, Japan) after calibration with water.

Parts of the concentrated lactose solutions were subsequently subjected to crystallization using a method previously proposed in [17]. The remaining portions of the concentrated solutions were analyzed to establish their properties. The pH of the samples was monitored using a pH meter (inoLab pH7110, WTW, Xylem Inc., Trifthof, Weilheim, Germany).

### 2.3. Crystallization Procedure

Rapid cooling of the concentrated solutions from 55 °C to 30 °C followed by a 2 h equilibration at the same temperature using a water bath was employed to induce nucleation, with immediate effect. This was followed by slow cooling at a rate of 5 °C/h until the solution temperature reached 15 °C. The solutions were stirred continuously and kept at a constant temperature (15 °C) throughout the night to achieve complete mutarotation. Once the solutions reached solubility equilibrium, the final solubility of all samples was determined by measuring the concentration of the supernatants. The lactose crystals were then separated through 0.45 µm filter paper and dried in an oven at 80 °C for four hours. The percentage yield of lactose was determined using the following equation:

$$Yield(\%)=\frac{0.95\times Mass\;of\;lactose\;crystals\;obtained}{Maximum\;theoretical\;crystal\;yield}\times 100$$

In order to accurately calculate the amount of lactose recovered, the crystallization of 50% *w*/*w* α-lactose monohydrate was taken into consideration. The maximum solubility of lactose, which was found to be 17.84 g of lactose in 100 g of water at a temperature of 15 ± 1 °C, was also considered. As a result, the maximum theoretical yield from a 50% solution was determined to be 82.16 g of lactose per 100 g of water. The detailed method used to calculate the crystal yield is explained elsewhere [17].

### 2.4. Differential Scanning Calorimetry (DSC)

Differential scanning calorimetry (DSC) serves as a fundamental method for quantifying the thermal characteristics of materials, enabling the establishment of a correlation between temperature and specific physical attributes of substances. Moreover, it stands as the sole approach for directly determining the enthalpy linked to the process under investigation. The thermal characteristics of the concentrated lactose solutions were measured immediately after concentration using a DSC instrument (DSC 1 STARe System, gas controller, GC 200, Mettler-Toledo, Columbus, OH, USA) combined with Mettler Toledo STARe evaluation software, version 15.00, according to the improved method [4] with slight modification to the scanning range. All samples were scanned with a starting temperature of 50 °C and end temperature of 200 °C at a heating rate of 5 °C/min. The onset and endset temperatures of water evaporation and related enthalpies (enthalpy is a measure of the total energy stored within a system) were analyzed using STARe thermal analysis software (Version 15.00) (Mettler Toledo).

Lactose crystal samples were also subjected to the same procedure with slight modifications. An amount of 2–5 mg of each crystal sample was scanned with a starting temperature of 50 °C and an end temperature of 250 °C at a rate of 10 °C/min following the method explained elsewhere [9].

### 2.5. Fourier-Transform Infrared Spectroscopy (FTIR)

The FTIR spectra of the concentrated lactose solutions and lactose crystal samples were collected using an FTIR spectrometer (Frontier 1, Perkin Elmer, Waltham, MA, USA). The concentrated solution spectra were collected instantly after reaching the solution concentration of ~55% (*w*/*w*) in the range of 4000–600 cm^−1^ in absorbance mode after background subtraction [14]. Every spectrum was obtained with an average of sixteen scans recorded at a resolution of 4 cm^−1^. Similarly, FTIR spectra of lactose crystal samples were also obtained in the range of 4000–600 cm^−1^ in absorbance mode, and the spectra were analyzed within the wavelength ranges of interest, 4000–2600 cm^−1^ and 1200–950 cm^−1^.

### 2.6. X-ray Diffraction (XRD)

X-ray powder diffraction patterns of all the samples were obtained using a Rigaku Miniflex 600 X-ray diffractometer (Rigaku Corporation, Wilmington, MA, USA). The lactose powder samples were firmly pressed to form a thin film (0.2 mm) on the glass sample holders before loading them into the instrument. The operating conditions were set at 40 kV and 15 mA and a step size of 0.02° with a speed of 1.2/min in a 2θ scanning range from 5° to 30° with a Ni Kβ-filter and a D/tex detector.

### 2.7. Statistical Analysis

Two replications of the entire experimental design were carried out. Observational data were analyzed with one-way ANOVA, and 95% confidence intervals were applied. Comparison of the means was assessed using Tukey’s test, with *p* < 0.05 considered statistically significant.

## 3. Results

### 3.1. pH Values of the Lactose Solutions in the Presence of Salts

The resulting pH of the pure lactose solution was found to be 5.9. The introduction of 22 mM Na into the lactose solution resulted in a decrease in pH to 5.6. The presence of 38 mM K and the cations combination of Mg^2+^, Ca^2+^, K^+^, and Na^+^ further reduced the pH to 5.5. The addition of 8 mM Mg and 30 mM Ca to the lactose solution resulted in further decreases in pH to 4.9 and 4.8, respectively (Table 2).

### 3.2. Crystal Yields and Final Solubilities of Lactose Solutions in the Presence of Salts

Pure lactose had a crystal yield of ~79%, which agreed with the results reported previously [9,17]. The final solubility of pure lactose was 17.6 g/100 g of water, which was close to a previously observed value of 17.84 g/100 g [18]. The presence of 8 mM of Mg or 22 mM of Na resulted in ~76% or ~75% crystal yields, respectively, with corresponding solubilities of 19 or 19.5 g/100 g of water. In the presence of 38 mM of K, the crystal yield decreased further to ~70%, which was followed by an increased solubility of 20.2 g/100 g of water. The resulting increased solubility and reduced yield of the lactose in the presence of KCl compared with PL is in agreement with a previous study [15]. Moreover, the presence of 30 mM of Ca yielded ~67% crystals with a resulting final solubility of 22.5 g/100 g of water. Further yield impairment was observed in the presence of cations in combination as the yield was further significantly reduced to ~63%, resulting in 28.5 g/100 g of water as the final solubility. The above-observed solubility results show an inverse relationship with the crystal yield (Table 3).

### 3.3. Thermal Behaviors of Concentrated Lactose Solutions and Crystals

The DSC analysis of the concentrated lactose solutions showed an endothermic peak at ~147 to ~155 °C, which was associated with the removal of water molecules from the lactose solution [19]. The recorded peak temperature was unaffected by the presence of 30 mM of Ca or 38 mM of K, while the presence of other cations individually and in combination slightly increased the peak temperature. However, the water removal peak temperatures were not significantly changed compared with pure lactose (Table 2). Similarly, the recorded onset and endset temperatures for water evaporation were also not affected (*p* > 0.05) by the presence of individual salts or salt mixture. In contrast, the enthalpy associated with water evaporation noticeably increased in the presence of different salts and the combination of salts. The average energy required to evaporate water from the pure lactose (PL) solution was ~698 J/g, while the addition of 22 mM Na, 38 mM K, 8 mM Mg, 30 mM Ca, and the salt mixture increased the average water evaporation enthalpy to ~918.6, 936.6, 987, 993.1, and 1005.4 J/g, respectively (Table 2). The correlation between the rise in the water evaporation enthalpy and the metal group, as well as the atomic radius of each group element, was noted in the present study. Na and K are classified in the alkali group, whereas magnesium (Mg) and calcium (Ca) belong to the alkaline group and show an increase in atomic radius with an ascending atomic number.

**Figure 1:**
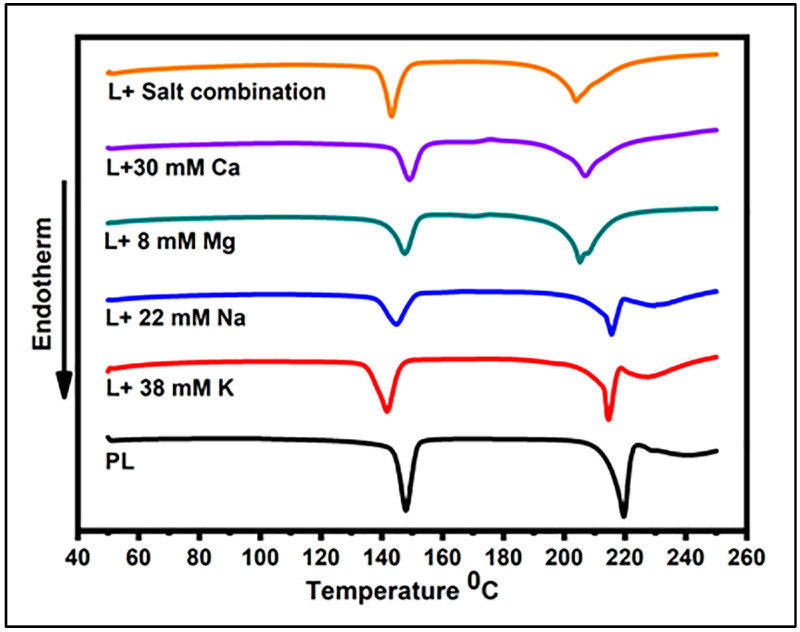
Dynamic DSC curves for lactose crystals in the presence of 8 mM Mg, 30 mM Ca, 38 mM K, 22 mM Na, salt combination, and pure lactose (PL).

The DSC thermograms of the lactose crystal samples are illustrated in Figure 1, with the parameters describing the thermal behaviors of the lactose crystals in the presence of salts shown in Table 3. In general, the temperature peaks in the DSC analysis were found to be associated with the presence or absence of amorphous lactose, crystalline lactose, and the related anomers (α and β forms) of lactose. The exothermic peak observed at ~167 °C is attributed to the transformation of amorphous lactose into its crystalline form. The endothermic peak detected at ~144 °C indicates the elimination of crystalline water, thereby providing evidence for the existence of the crystalline form of lactose. The α and β forms of lactose can be identified by the presence or absence of α-lactose and β-lactose melting peaks at approximately 213 °C and 224 °C, respectively [20,21]. All the samples in the present study were characterized by an endothermic peak at approximately 140–150 °C, revealing the crystalline nature of the samples. This peak refers to the loss of water molecules from a crystal structure [19,22]. The resulting peak values for 22 mM Na, 38 mM K, and salt mixture were significantly different (*p* < 0.05) from that of PL. On the other hand, the peak values for 8 mM Mg or 30 mM Ca were not significantly different from that of PL (Table 3). The DSC thermograms for the lactose crystals in the presence of 30 mM Ca, 8 mM Mg, and 22 mM Na exhibited a small exothermic peak at ~175 °C, which could be associated with the recrystallization of amorphous lactose present in the relevant samples [23]. The PL crystals had the greatest enthalpy value for the removal of crystalline water (167 J/g), which was significantly diminished in the presence of salts (*p* < 0.05). The greatest decline was observed in the presence of a salt mixture, resulting in an enthalpy value of 129.6 J/g, which presents a ~23% decrease compared with that of PL. When it came to individual salts, the magnitude of reduction was the greatest with monovalent cations with a ~20.2% and 20% decline in the presence of 22 mM Na and 38 mM K. At the same time, 30 mM Ca and 8 mM Mg decreased the enthalpy by 17% and 10.9%, respectively. All the lactose samples exhibited another endothermic peak at ~200–220 °C, which can be assigned as the melting of α-lactose [24]. An additional endothermic peak was observed subsequent to the α-lactose melting peak at approximately 228 °C in the PL sample and samples containing Na and K. This additional peak is likely attributed to the melting of β-lactose [25]. This β-lactose peak disappeared in the DSC thermograms of lactose samples containing the salt mixture, Ca, or Mg.

### 3.4. FTIR Spectra of Concentrated Lactose Solutions and Crystals

**Figure 2:**
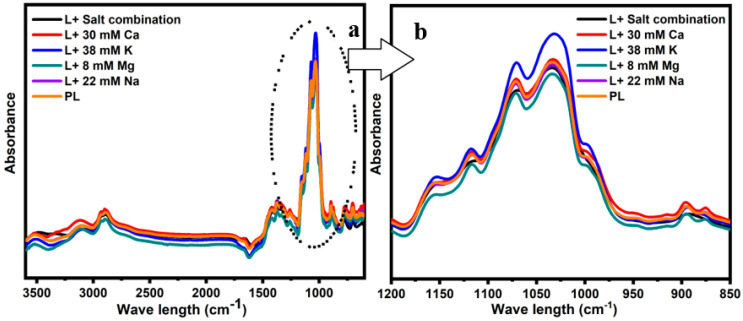
FTIR spectra obtained from the concentrated lactose solutions in the presence of 8 mM Mg, 30 mM Ca, 38 mM K, 22 mM Na, the salt combination, and pure lactose (PL) in the ranges of 4000–600 cm^−1^ (**a**) and 1200–800 cm^−1^ (**b**).

The behavior of water molecules within a food system is dependent on various interactions taking place between components of the food matrix. As such, understanding how water molecules behave within a food system is crucial to controlling the stability of food systems and crystallization. FTIR is a simple and rapid technique that can be used to determine these changes via the molecular vibrations of various compounds in the food system. In Figure 2 (range 4000–600 cm^−1^), Figure 2a represents the FTIR spectra obtained for the concentrated lactose solutions in the presence of various cations. The physical state of carbohydrates was determined by analyzing the vibrations and stretching of C-C and C-O bonds in the 800–1200 cm^−1^ range [26]. Figure 2b illustrates the FTIR spectra of lactose with or without the addition of individual salts and their mixture in the range of 1200–850 cm^−1^. Two strong peaks were observed in all concentrated lactose solutions at ~1030 cm^−1^ and 1070 cm^−1^. It was found that the corresponding peaks were correlated with C-C stretching vibrations and C-O stretching in lactose, respectively [27,28]. The greatest intensity of these peaks was observed in the presence of 38 mM K, indicating greater stretching vibrations in the lactose molecules, while the lowest was observed for the lactose solution containing 8 mM Mg, which could be attributed to the restricted movements of the lactose molecules. No considerable change, relative to the control, was observed in the intensity of the 1200 to 850 cm^−1^ range for the samples with 30 mM Ca, 22 mM Na, or the salt mixture.

**Figure 3:**
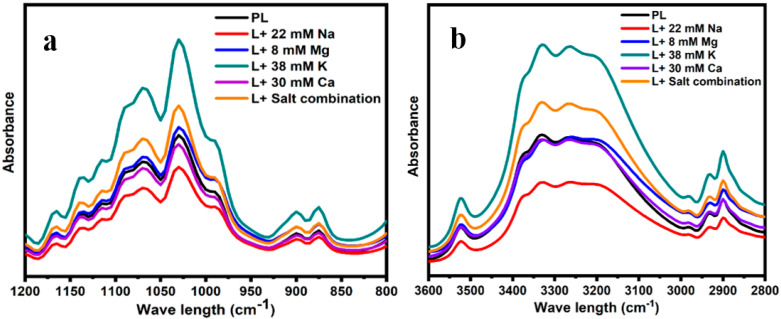
FTIR spectra obtained from the lactose crystals in the presence of 8 mM Mg, 30 mM Ca, 38 mM K, 22 mM Na, the salt combination, and pure lactose (PL) in the ranges of 3600–2800 cm^−1^ (**b**) and 1200–800 cm^−1^ (**a**).

The behaviors of lactose crystals determined using FTIR are shown in Figure 3a,b. The spectral region of 1200–800 cm^−1^ in Figure 3a can be used to differentiate the crystalline form from amorphous lactose [21]. More defined sharp peaks in this range are associated with crystalline lactose, while indistinct peaks symbolize amorphous lactose [9]. All the studied samples in this study were characterized by sharp peaks in the corresponding region, indicating the crystalline nature of lactose in the presence of different salts.

The spectral region between 3600 and 2800 cm^−1^ in Figure 3b shows the behaviors of water molecules and H bonding within the crystal lattices in different hydration states [29]. All samples showed broad bands of vibration corresponding to the intermolecular stretching vibrations of the hydroxyl group [23,30]. However, the deviation in these stretching vibrations appeared to depend on the type of salt present in the samples. The sample containing a concentration of 30 mM Ca, followed by 8 mM Mg, the salt mixture, and 38 mM K, exhibited the most significant increase in the intensity within the region of 3600–2800 cm^−1^ when compared with the reference sample (PL). Conversely, the lactose crystals obtained with a concentration of 22 mM Na displayed the lowest intensity in this region. Furthermore, the shape and location of the sharp, distinct O-H stretching peak at 3520 cm^−1^ [30] are indicative of constrained water molecules in the crystal lattice. This peak is more prominent in the presence of 38 mM K but, in contrast, diminished in the presence of 22 mM Na.

### 3.5. XRD Patterns of the Lactose Crystals in the Presence of Salts

**Figure 4:**
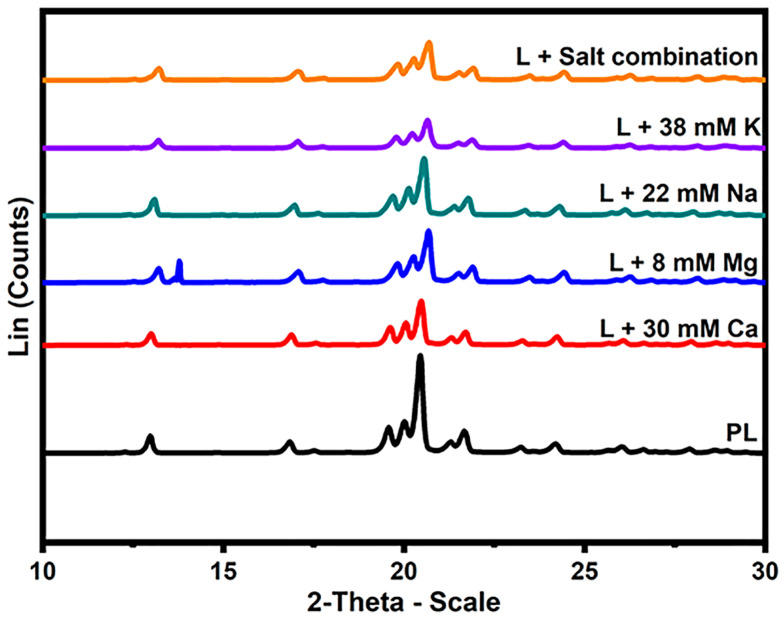
X-ray diffraction patterns of lactose crystals with the addition of 8 mM Mg, 30 mM Ca, 38 mM K, 22 mM Na, salt combination, and pure lactose (PL).

The XRD technique was used to identify the distinct polymorphic forms of lactose in the presence of different salts (α and β) and determine the purity of the lactose powders. Figure 4 shows the characteristic XRD diffractograms of lactose crystals with or without the addition of different salts. The positioning of the peaks (2θ) denotes the distinct varieties of lactose crystals that are present within the sample. For example, α-lactose is discernible at 12.5°, 16.4°, 20.0°, and 20.1°, while anhydrous β-lactose is identifiable at 10.5°, 19.0–19.5°, 20.9°, and 21.0°. Several additional peaks are attributed to the combinations of lactose crystals, including anhydrous α:β with a molar ratio of 5:3 at 19.1° and 21.1°, as well as anhydrous α:β with a molar ratio of 4:1 at 19.5° [30,31,32]. In the present study, the crystallization of lactose into the α-lactose monohydrate form is discernible in the peaks observed at 12.9–13.2°, 16.8–17.0°, 20.1°, 20.3°, and 20.5° (as presented in Table 4) in nearly all the samples that were examined.

Crystallization of lactose into anhydrous β-lactose was detected in the PL and the samples containing monovalent cations (Na and K) at 19.5°, 20.8°, and 20.6°, respectively, which closely correlate with the diffraction angles reported in the literature (Table 4). Moreover, the samples obtained in the presence of 22 mM Na and 38 mM K resulted in peaks with diffraction angles of 21.4° and 21.3°, which could be related to the mixture of anhydrous α:β with a molar ratio of 5:3. The minor variances observed in the diffraction angles in the current investigation, when contrasted with the literature [33], may be attributed to the augmented interlayer spacing of crystals resulting from the interaction of salts with water and lactose.

## 4. Discussion

Several factors affect the crystallization of lactose, including impurities, super-saturation, temperature, viscosity, lactose concentration, and pH [34]. Based on the results shown in the tables and figures, lactose crystallization behavior is impacted by the presence of cations in a varying manner depending on the type and likely concentration. The process of lactose crystallization typically consists of two fundamental stages: “nucleation” and “crystal growth” [35]. During crystal growth, the enlargement of nuclei occurs in response to the prevailing level of supersaturation in the solution. Cations as impurities in lactose solutions govern lactose crystallization in a direction that is dependent on the concentration and the type of cation present [8] as it induces alterations in solubility. Alterations in solubility have a direct impact on the state of supersaturation within the solution, consequently influencing the processes of nucleation and crystal growth [36].

Potassium (K^+^) is an ion that disrupts the structure of water and has been found to influence the orientation of water molecules [36]. This, in turn, enhances the solubility of lactose and reduces lactose–lactose interactions, disrupting the orderly arrangement of lactose, which results in the hindrance of lactose crystal growth. In contrast, it has been observed that Na^+^ ions have the ability to decrease the solubility of lactose when combined with phosphate [37]. Nevertheless, the present investigation revealed that the inclusion of NaCl as an additive led to an increase in solubility when compared with PL. However, the proportional increase in solubility was lower than that observed with KCl, which resulted in a higher yield. This can be attributed to the fact that NaCl is a more soluble salt than KCl and thus possesses a greater water-binding capacity [37], which ultimately leads to a reduction in lactose solubility when compared with KCl. Ions with a very high charge density electrostatically organize water effectively. Ca^2+^ is a divalent ion with a strong electric field, which acts as a water structure promoter [38]. Therefore, the alteration in water structure to varying extents might affect the solubility of lactose and the supersaturation of the solution, and, thereby, the crystal yields. Mg^2+^, on the other hand, creates lactose-Mg complexes, increasing the solubility of lactose [39]. However, this effect is much lower than the effect of Ca^2+^ on the solubility and crystallization of lactose (Table 3).

Lactose, being a hydrophilic molecule, is enveloped by a hydration layer [4]. Consequently, during nucleation, the water molecules in the hydration layer of lactose undergo de-solvation, thereby enabling lactose–lactose interactions that culminate in the creation of the crystal lattice. The presence of a cation may enhance the bonding between these hydration water molecules and the lactose molecules, thereby impeding the evaporation of these water molecules. This is demonstrated by an increase in water evaporation enthalpies in the presence of different cations, as shown in Table 2. In this table, the greatest enthalpy of 167 J/g for the loss of crystalline water from crystal lattice was obtained for the PL. All other measured enthalpies obtained in the presence of different cations as well as their combination were reduced, indicating the obstruction of water removal from the crystal lattices [40].

Ca^2+^ ions strongly interact with four to six layers of water molecules via dipole–ion interactions [41]. Hence, the water molecules may be densely packed within these hydration layers in comparison with water molecules in the pure lactose solution, leading to restricted mobilities, which may be applied to other cations as well. Plausibly, individual lactose molecules may be surrounded by a strong hydration layer, creating a shielding effect and hindering water removal from the crystal lattice. Interestingly, two protruding peaks initiating at ~1030 cm^−1^ and 1070 cm^−1^, which are associated with the OH groups of lactose, C-C stretching vibrations, and C-O stretching in the glucose molecule of lactose [27], exhibited significant reductions in intensity in the presence of 22 mM Na. This indicated the restricted movement of the lactose molecules due to the formation of a strong hydration layer around them. The mechanisms by which each individual cation and/or in combination appeared fundamentally different from each other. Cations affect both nucleation and crystal growth rates depending on the radius/charge ratio of the cation due to its hydrophilic and hygroscopic nature [42]. Mg^2+^ has the lowest radius/charge ratio and, thus, a high binding capacity to water. On the other hand, Ca^2+^ has the greatest radius/charge ratio and, consequently, a small binding capacity compared with Mg^2+^. The lowest yield of lactose crystals was obtained in the presence of Ca^2+^ compared with that in the presence of Mg^2+^, which was close to that of pure lactose. This indicates that the binding capacity of Mg^2+^ with water allows more lactose molecule interactions and, thereby, crystallization compared with Ca, although this effect cannot be ruled out completely. Furthermore, the incorporation of a cation onto the surface of an existing crystal increases the surface potential difference as a result of the newly charged crystal surface. This, in turn, decreases the thickness of the diffusion layer and, consequently, leads to the acceleration of the diffusion step of the crystallization and, thus, the yield. This effect may have an increased influence in a high-charge-density cation such as Ca^2+^. In addition, Ca associations with lactose crystals have been found to be enhanced in the presence of other cations [15]. Thus, the further decreased crystal yields in the presence of the salt mixture compared with the presence of Ca may be partly due to the incorporation of the cations into the lactose molecules. This incorporation of cations may also increase the distances between lactose molecules and delay the required orderly arrangement of lactose molecules in forming a crystalline structure.

Previously, it was hypothesized that pH is an important factor in the crystallization of lactose [43]. By altering the pH, the mutarotation of lactose can be accelerated, which favors crystallization [34]. In the presence of all cations, the pH declined, which could have partially played a part in crystallization (Table 2). However, the sample with 8 mM Mg^2+^ had a pH value close to that of the solution containing 30 mM Ca^2+^, which also indicates that pH could not be solely responsible for the crystallization patterns. In addition, further research is needed to establish an exact mechanism outlining lactose crystallization as a function of pH.

Water molecules within a crystal lattice can reside either in the lattice, lattice channels, or ion-coordinated sites [9]. Hydrogen bonds are sensitive to charge transfer from donors to acceptors [34]. Water molecules residing in lattice sites are isolated from other water molecules due to contact with lactose, while water molecules residing in lattice channel sites are in contact with other water molecules of adjoining unit cells [44]. These water molecules can interact via H-bonding and ion-coordinated bonds. Ion-coordinated water bonds are thermodynamically preferred and stronger than hydrogen bonds. Thus, the extent of the formation of H bonds and ion-coordinated bonds within these water molecules in the crystal lattice in the presence of different cations may change the behavior of the lactose crystals. The present study clearly showed the changes in H-bonding patterns in the presence of Ca (Figure 3) compared with those in the presence of other cations. However, the presence of the salt mixture showed much greater intensities within the H-bonding region (Figure 3) compared with Ca, highlighting the fact that cations in combination may influence the balance between these two types of bonds and thereby change the lactose crystallization behavior.

Another interesting finding of the current study is that the β-lactose melting peak disappeared in the DSC thermograms of lactose in the presence of 30 mM Ca, 8 mM Mg, and the salt mixture. The disappearance of the β-lactose melting peak may be a direct result of the prevention of lactose isomerization, which can be attributed to the presence of Ca^2+^ and/or Mg^2+^. The impact of Ca and Mg on the inhibition of lactose isomerization or the impact of mutarotation equilibrium is still unclear as there is no established evidence in the literature. However, this result was further supported by the observed XRD diffraction angles for β-lactose in the presence of Na^+^, K^+^, and PL. The aforementioned findings underscore the importance of water molecule behavior and inter- and intra-molecular interactions in governing the behavior of lactose. Overall, the impact of monovalent salts on the crystallization behavior of lactose was found to be comparatively less influential than that of bivalent salts. The change in crystallization behavior can be modulated by adjusting the concentrations of cations present in acid whey. Additional investigations are warranted to assess the behavior of lactose under varying cation concentrations and the impact of anions in acid whey.

## 5. Conclusions

The process of isolating lactose from a complex mixture like acid whey necessitates precise processing conditions. The formation of lactose crystals and their growth are influenced by the characteristics and actions of the solvent and water, which are significantly affected by the presence of other substances. Crystallization is influenced by the presence of different cations. The presence of the salt mixture containing 8 mM Mg, 30 mM Ca, 38 mM K, and 22 mM Na had the greatest impact on the lactose crystal yield compared with that of pure lactose. This can be attributed to the increased interactions between lactose and cations, resulting in changes in solubility and the structure of water molecules, ultimately affecting the crystallization behavior of lactose. In the presence of all cations, pH declined, which could partially play a part in crystallization. Divalent ions impacted the behavior of lactose by altering the anomerization of lactose as opposed to the monovalent ions. The present study highlighted the fact that the presence of Ca plays a major role in the crystallization of lactose. However, further research is needed to evaluate the effect of the presence of various anions on lactose crystallization.

## Figures and Tables

**Table 1:** Combinations of lactose and salt used in this study.

Lactose Salt Combination	Composition of Each Combination
Pure lactose	5 g/100 g solution
Lactose + KCl	5% lactose + 38 mM K
Lactose + CaCl_2_	5% lactose + 30 mM Ca
Lactose + NaCl	5% lactose + 22 mM Na
Lactose + MgCl_2_	5% lactose + 8 mM Mg
Lactose + salt mixture	5% lactose + 38 mM K + 30 mM Ca + 22 mM Na + 8 mM Mg

**Table 2:** pH values; onset, peak, and endset temperatures; and enthalpy values for loss of water in concentrated lactose solutions, as affected by the presence of different cations in the form of chlorides.

Sample	pH *	Onset Temperature (°C)	End Set Temperature (°C)	Peak Temperature (°C)	Enthalpy Water Evaporation (J/g)
Pure lactose	5.9	143.8 ± 9.1 ^a^	154.5 ± 10.5 ^a^	147.6 ± 9.4 ^a^	698.4 ± 6.5 ^d^
Lactose + KCl	5.5	147.3 ± 3.9 ^a^	158.2 ± 2.3 ^a^	151.8 ± 1.6 ^a^	936.6 ± 29.8 ^bc^
Lactose + CaCl_2_	4.8	143.2 ± 3.4 ^a^	155.2 ± 0.4 ^a^	148.2 ± 1.2 ^a^	993.1 ± 30.8 ^a^
Lactose + NaCl	5.6	148 ± 2.9 ^a^	165.2 ± 1.1 ^a^	156.8 ± 1.6 ^a^	918.6 ± 4.6 ^c^
Lactose + MgCl_2_	4.9	146.4 ± 0.0 ^a^	164.9 ± 0.0 ^a^	155.3 ± 0.0 ^a^	987 ± 0.0 ^ab^
Lactose + salt mixture	5.5	154.2 ± 1.1 ^a^	163.8 ± 0.9 ^a^	155.2 ± 1.0 ^a^	1005.4 ± 15.5 ^a^

* SD for pH was ± 0.05. ^a–d^ Values in the same column with different superscript letters are significantly different (*p* < 0.05).

**Table 3:** Mean crystal yields, solubilities; onset, peak, and endset temperatures; and enthalpy values for crystallization and melting points of lactose crystals, as affected by the presence of different cations in the form of chlorides.

Sample	Mean Crystal Yield (%)	Solubility (g/100 g of Water)	Onset Loss of Crystalline Water (°C)	Peak Loss of Crystalline Water (°C)	Endset Loss of Crystalline Water (°C)	Enthalpy of Loss of Crystalline Water (J/g) *	α-Lactose Melting Peak (°C)	β-Lactose Melting Peak (°C)
Pure lactose	79	17.6	143.3 ± 0.8 ^ab^	147 ± 0.4 ^a^	152.9 ± 1.4 ^a^	167 ± 8.6 ^a^	219.5 ± 0.9 ^a^	230.5 ± 0.9 ^b^
Lactose + KCl	70	20.2	137.2 ± 1.3 ^d^	141.2 ± 0.9 ^c^	145.2 ± 1.1 ^d^	133.3 ± 4.9 ^c^	213.6 ± 0.6 ^b^	228.5 ± 0.6 ^a^
Lactose + CaCl_2_	67	22.5	144.5 ± 0.3 ^a^	148.1 ± 0.9 ^a^	154.4 ± 1.2 ^a^	138.1 ± 3.8 ^bc^	207.3 ± 0.8 ^c^	ND
Lactose + NaCl	75	19.5	138.4 ± 0.8 ^cd^	143.9 ± 0.7 ^b^	148.8 ± 1.3 ^bc^	133.2 ± 0.9 ^c^	214.6 ± 0.6 ^b^	230.7 ± 1.4 ^b^
Lactose + MgCl_2_	76	19.0	142.1 ± 0.2 ^b^	146.5 ± 0.7 ^a^	151.6 ± 0.7 ^ab^	148.8 ± 0.2 ^b^	205.1 ± 0.3 ^d^	ND
Lactose + salt mixture	63	28.5	139.7 ± 0.2 ^c^	142.8 ± 0.2 ^bc^	148.0 ± 1.4 ^cd^	129.7 ± 8.5 ^c^	203.3 ± 0.5 ^e^	ND

* Enthalpy refers to the area under the peak obtained using DSC. Values are presented as means of three individual observations (3 ≥ *n*) plus or minus standard deviation. ^a–e^ Values in the same column with different superscript letters are significantly different (*p* < 0.05). ND = not detected.

**Table 4:** Characteristics of the different forms of lactose crystals observed in XRD diffractogram patterns at various diffraction angles (2θ)°.

Types of Crystals	Diffraction Angles (2θ)° (Literature Data) *	Measured Diffraction Angle (2θ)°					
		Pure Lactose	Lactose + CaCl_2_	Lactose + MgCl_2_	Lactose + KCl	Lactose + NaCl	Lactose + Salt Mix
α-lactose monohydrate	12.5	12.9	13.0	ND	13.0	13.0	13.2
	16.4	16.8	16.8	17.0	16.9	16.8	17.0
	20.0/19.9	ND	ND	ND	ND	ND	ND
	20.1	20.3	20.4	20.1	ND	20.5	ND
Stable anhydrous α-lactose	19.37	ND	ND	ND	ND	ND	ND
Anhydrous β-lactose	10.5 19.0 20.9 21.0	19.5	ND	ND	20.6	20.8	ND
Anhydrous, α:β molar ratio 5:3	19.1	ND	ND	ND	ND	ND	ND
	21.1	ND	ND	ND	21.3	21.4	ND
Anhydrous, α:β molar ratio 4:1	19.5	19.5	ND	ND	ND	ND	ND

* Literature data [30,31,32]; ND = not detected.

## Data Availability

Data are contained within the article.
